# DEM-CFM analysis of Lode angle influence on the undrained cyclic behavior of anisotropically consolidated soils

**DOI:** 10.1038/s41598-025-02650-3

**Published:** 2025-06-02

**Authors:** Mohammad Salimi, Nazanin Irani, Torsten Wichtmann

**Affiliations:** Chair of Soil Mechanics, Foundation Engineering, and Environmental Geotechnics, Ruhr-University Bochum, Bochum, Germany

**Keywords:** DEM-CFM, Anisotropic consolidation, True triaxial test, Undrained cyclic, Lode angle, Civil engineering, Theoretical particle physics

## Abstract

This study examines the behavior of anisotropically consolidated granular assemblies under undrained cyclic true triaxial loading paths. To achieve this, the Discrete Element Method (DEM) is conjugated with the Coupled Fluid Method (CFM) to account for fluid-solid interaction in undrained conditions. The examined loading paths include two phases: anisotropic consolidation and undrained cyclic true triaxial loading. During consolidation, samples are sheared at various Lode angles to reach a spectrum of initial static shear stress levels. In the second stage, undrained cyclic loading is applied with constant shear stress amplitudes at various Lode angle values. The results indicated that the monotonic and cyclic Lode angle, initial static shear stress, and amplitude of deviatoric stress have pronounced effects on the secant shear modulus degradation and the rate of excess pore water pressure generation of granular assemblies. In tandem with macro-scale observations, the evolution of the microstructure within assemblies is analyzed using the coordination number, redundancy index, inter-particle contact fabric tensor, and particle orientation fabric tensor. The micro-scale findings confirm that the anisotropy induced by changes in the loading direction significantly impacts the shear strength of the assemblies. Additionally, the fabric of assemblies aligns along the preferential direction corresponding to the major principal stress, influencing the dilative response.

## Introduction

The cyclic response of granular soil under general loading paths is of significant interest in geotechnical engineering, as many civil infrastructures are subjected to multidirectional loads from earthquakes, traffic, and tidal waves^1,2^. Importantly, in-situ soil deposits frequently undergo anisotropic stress conditions, characterized by unequal principal effective stresses. It has been well-documented that the mechanical behavior of soils is markedly altered when cyclic deviatoric stress is applied following an initial static shear stress, which refers to anisotropic consolidation^3–7^. Thus far, numerous studies have examined the impact of anisotropic consolidation on the cyclic shear behavior of soils in experimental setups (among all,^8–11^). Acknowledging the extensive research in the field, most laboratory element-level tests are focused on loading under axisymmetric conditions, *e.g.*, triaxial, or in particular simple shear, which may not account for the effects of anisotropic loading conditions and intermediate stress. Moreover, available research on anisotropically consolidated granular soils often assumes that cyclic loading applies in the same direction as the initial static shear stress is imposed during the consolidation. This assumption may not fully capture the complexities of real-world loading scenarios, where soils might undergo undrained cyclic loading with a loading direction that differs from that of the initial pre-shearing phase. The experimental studies also highlighted that the deformation and shear strength of soils are heavily influenced by the direction of the initial static shear stress, which is mainly associated with the anisotropic nature of the samples^12,13^. For instance, using monotonic and cyclic triaxial tests,^12^ indicated that the cyclic strength of the soil varies depending on whether the initial shear stress is applied in the triaxial extension mode or the triaxial compression mode. Hence, it is necessary to investigate the response of anisotropically consolidated soils under general stress paths. To study the effects of stress paths aligned with different Lode angles on soil behavior, specialized testing equipment such as true triaxial or hollow cylinder devices is required, which are available mainly in advanced laboratories. Additionally, analyzing microstructural evolution during the loading process is not feasible in a typical experimental setup due to the need for specialized imaging techniques, such as x-ray tomography^14^, photoelasticity^15^, and stereophotogrammetry^16^. An alternative approach is to use the DEM to explicitly simulate the interactions between individual particles under general and anisotropic loading paths. In the realm of DEM simulations, the majority of studies investigating the behavior of anisotropically consolidated samples under cyclic loading have primarily focused on drained triaxial and true triaxial paths (e.g.,^4,17^), as well as undrained triaxial conditions (e.g.,^6,18–23^). However, examining the undrained cyclic response of anisotropically consolidated samples subjected to different Lode angles remains a significant gap in the current body of knowledge, and this study aims to provide insights into this research topic. Various methods have been employed for simulating the undrained behavior using DEM. The most prevalent approach is the constant volume method (CVM), which restricts any changes in the total volumetric strain of the particulate assemblies. Another strategy involves integrating DEM with an additional numerical approach, such as Computational Fluid Dynamics (CFD) or Smoothed-Particle Hydrodynamics (SPH), to incorporate the effects of pore fluid in the analysis. However, this combined approach can be computationally intensive^24^. To address this, CFM approaches have been developed to account for the interaction between solid particles and fluid by calculating pore water pressure at the mesoscopic scale (e.g.,^24,25^). Generally, Darcy’s law is used to computationally model the fluid phase and the permeability is defined based on the geometric, chemical, physical, and mechanical properties of the medium^26^. Studies such as^24,25,27^ and^28^ have shown that the DEM-CFM coupling approach is effective in simulating the behavior of granular assemblies under different stress conditions. In the work of^29^, the proposed DEM-CFM coupling scheme was initially validated by comparing its simulations with CVM results under monotonic triaxial compression, while^30^ provided additional validation under undrained cyclic triaxial conditions. Following validation under axisymmetric conditions, a comprehensive series of true-triaxial tests with varying Lode angles was conducted. Beyond the advantage that CFD and CFM methods offer more physically accurate simulations of undrained conditions through explicit fluid-particle interaction modeling, the CVM may present limitations for stress-controlled path simulations. The primary challenge lies in maintaining constant volume conditions under stress-controlled loading, particularly when stress and strain tensors are non-coaxial. This effect becomes more pronounced when fabric anisotropy is taken into account^31^. Given that this study involves stress-controlled loading paths and inherent stress-strain non-coaxiality under true triaxial loading conditions, the conventional constant volume approach proved unsuitable for our research objectives. Consequently, this study implements the DEM-CFM coupling approach.

The primary aim of this work is to explore the impact of the Lode angle on the undrained cyclic behavior of anisotropically consolidated samples. To achieve this, the DEM-CFM coupling scheme is utilized. Initially, the particulate assemblies undergo anisotropic consolidation (*say* pre-shearing phase), followed by undrained cyclic true triaxial loading. The influence of the Lode angle during both consolidation and cyclic shearing, along with the effects of initial static stress and deviatoric stress amplitude on the mechanical behavior of granular assemblies are thoroughly examined. Subsequently, the microstructural evolution of the assemblies is analyzed using coordination number, redundancy index, inter-particle contact orientation, and particle orientation fabric tensors. These micro-scale observations are then employed to elucidate the macro-scale responses.

## Numerical procedure

This study employs the 3D Particle Flow Code (PFC3D)^32^ to simulate the behavior of granular assemblies under undrained cyclic loading. Using the Fish programming language, the DEM is conjugated with the CFM to simulate undrained conditions. CFM employs Darcy’s law to model solid-fluid interactions, assuming that all pores are interconnected. The following subsections provide short descriptions of the governing equations and numerical procedures. A comprehensive derivation and detailed explanation of the CFM formulation can be found in^27–30^. Given that this method has been thoroughly validated and calibrated in these prior works, this study will employ the same set of parameters and methodology.

### The DEM-CFM coupling scheme

In general, the DEM-CFM coupling scheme comprises two primary components: the solid phase and the fluid phase. The solid phase dictates the behavior of the solid grains within the assembly, whereas the fluid phase manages the voids that affect pore water pressure fluctuations. In this study, the assemblies are fully saturated, meaning all voids are filled with water, representing the pore-fluid phase. To accurately describe the solid and fluid phases, it is essential to present the governing equations related to the stress tensor and changes in pore pressure increments.

To calculate the true effective stress tensor at the macroscale, the following equation is utilized^33^:

1 $$\begin{aligned} \varvec{\sigma }^\prime = \frac{1}{V}\sum _{c=1}^{N_{c}} \textbf{l}^{c} \otimes \textbf{f}^{c} \end{aligned}$$

wherein *V* indicates the volume of the assembly, $N_{c}$ denotes the number of particle-to-particle contacts, $l^{c}$ and $f^{c}$ are the branch vector and force at contact *c*, respectively. $\otimes$ is the dyadic product of two vectors. The mean effective stress is calculated as $p^\prime =\sigma ^\prime _{ii} / 3$ and deviatoric stress is determined as $q=\sqrt{3\sigma ^*_{ij}\sigma ^*_{ij} / 2}$. $\sigma ^*_{ij} = \sigma ^\prime _{ij} - \delta _{ij} p^\prime$ describes the deviatoric component of the stress tensor, with $\delta _{ij}$ representing the Kronecker delta ($\delta _{ij}$, equals 1 when $i = j$ and 0 when $i \ne j$). The Lode angle ($\theta$) is defined as:

2 $$\begin{aligned} cos(3\theta ) = \dfrac{J_3}{2}\left( \dfrac{3}{J_2}\right) ^2 \end{aligned}$$

where $J_2=\Vert \sigma ^*_{ij}\Vert$ and $J_3=$det$(\sigma ^*_{ij})$ are defined. det$(X_{ij})$ denotes the determinant of the tensor $\varvec{X}$. In this study, the Lode angle is associated with the stress increment.

CFM treats the fluid phase as a continuum, where changes in void volume are related to the evolution of pore pressure, assuming a uniform distribution within each measurement region. Under undrained conditions, previous studies have shown that drag, capillary, and buoyancy forces are negligible compared to the excess pore pressure generated during shearing^34^. This allows for a simplified formulation in which the pore pressure is uniformly distributed across the particle assembly. In the DEM-CFM coupling scheme, the unbalanced force acting on particles located at the boundary of a measurement region is computed by considering pressure changes due to both fluid inflow/outflow and volumetric deformation. The pressure change associated with fluid exchange between neighboring measurement regions, $\Delta P_f$, is calculated based on Darcy’s law:

3 $$\begin{aligned} \Delta P_f = \frac{E_w}{nV} \sum \Delta Q \end{aligned}$$

where $E_w$ is the bulk modulus of water, *n* is the porosity, *V* is the volume of the measurement region, and $\sum \Delta Q$ denotes the net fluid flux.

The pressure change due to local changes in void ratio, $\Delta P^{\prime }_f$, is computed using:

4 $$\begin{aligned} \Delta P^{\prime }_f = E_w \, \varepsilon _v \end{aligned}$$

where $\varepsilon _v$ is the volumetric strain.

The total unbalanced force, $F^{\text {unbalanced}}$, acting on the boundary particles is then given by^29^:

5 $$\begin{aligned} F^{\text {unbalanced}} = \left( P_m + \Delta P^{\prime }_f + \Delta P_f\right) A \end{aligned}$$

where $P_m$ is the mean pressure in the measurement volume and *A* is the area over which the force is applied.

### Sample preparation and test procedure

**Fig. 1:**
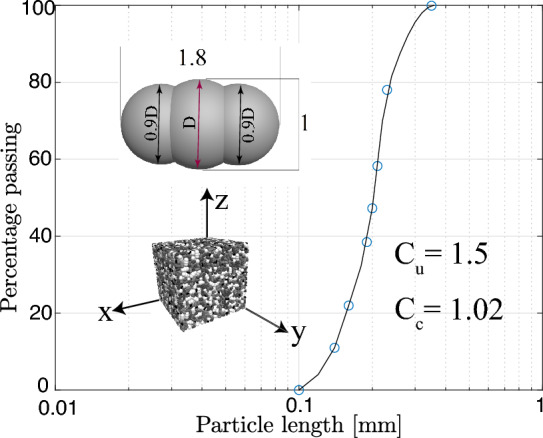
Particle size distribution curve and general shape of particles.

Simulations were conducted on isotropically consolidated samples composed of non-spherical, elongated particles, as illustrated in Fig. 1. These particles have an aspect ratio of 1.8:1, with diameters optimized using the methodology proposed by^35^. An aspect ratio of 1.8:1 was chosen based on the median value reported by Mitchell et al.^36^, p. 88], representing a balance between the sub-angular characteristics of sand particles (aspect ratio 1.0:1.5) and the more elongated shapes typical of silt particles (1.5–3.0). The simulations employed a poorly graded sand, characterized by the Particle Size Distribution (PSD) curve shown in Fig. 1, with a coefficient of uniformity $C_u = 1.5$ and coefficient of curvature $C_c = 1.02$.

Given the elongated shape of the particles, and following the suggestion of^37^, a rigid wall condition with zero friction on the boundary walls was applied^38,39^. The simulation utilized a linear contact model, assuming uniform and well-connected particles in the granular media. Material deformation under small strains was described using an isotropic material model based on elastic constants, such as Young’s modulus. For the silica sand, with an effective Young’s modulus of approximately 10.60–12.70 MPa^40,41^, a reference stiffness of $10^5$ kPa was assumed for samples under a mean effective stress of 100 kPa^42,43^. The tangential and normal contact stiffness values are assumed equal for simplicity-an approach commonly applied to non-cohesive, sub-rounded particles as considered in this study (e.g.,^44–46^). The bulk modulus of pore water ($E_w$) was set at 100 MPa to ensure negligible volume changes (less than $10^{-6}$) during shearing, balancing simulation efficiency and accuracy. While a higher $E_w$ value would yield similar macroscopic outcomes, it would require smaller time steps to maintain quasi-static system behavior. For sub-angular sand with an internal friction angle of approximately $30^\circ$, an inter-particle friction coefficient ($\mu$) of 0.5 was adopted^43,47^. To ensure quasi-static loading conditions, the applied shear strain rate was kept below $5 \times 10^{-5}~\text {s}^{-1}$, in accordance with the criterion proposed by^48^. Additionally, the simulations satisfied the inertial number criterion, maintaining $I < 0.001$ as recommended by^49^. The inertial number *I*, defined as (^50^):

6 $$\begin{aligned} I = \frac{\dot{\gamma } d}{\sqrt{p / \rho }} \end{aligned}$$

where *I* quantifies the ratio of inertial to confining forces, where $\dot{\gamma }$ is the shear strain rate, *d* is the particle diameter, *p* is the confining pressure, and $\rho$ is the particle density.

Accordingly, a strain rate of $5 \times 10^{-5}~\text {s}^{-1}$ and a time step of $1.5 \times 10^{-7}~\text {s}$ were selected for the simulations. A summary of all simulation parameters is provided in Table 1.

**Table 1:** Parameters used in the simulations.

	Parameter	Value
DEM	Particle density ($\rho _{p}$)	2650 kg/m^3^
	Inter-particle friction coefficient ($\mu$)	0.5 (−)
	Damping ratio	0.7 (−)
	Wall-particle friction coefficient	0.0 (−)
	Wall stiffness	10^7^ kN/m
	Normal contact stiffness ($k_{n}$)	$k_{0}^{*} \cdot r$
	Tangential contact stiffness ($k_{s}$)	$k_{n}$
	Number of clumps (pebbles)	9100 (27300)
CFM	Bulk modulus of pore-fluid ($E_{w}$)	100 MPa
	Pore-water density ($\rho _{w}$)	1000 kg/m^3^

$$^*k_{0} = 10^8$$ N/m^2^

### Implementation of monotonic and cyclic loading conditions

**Fig. 2:**
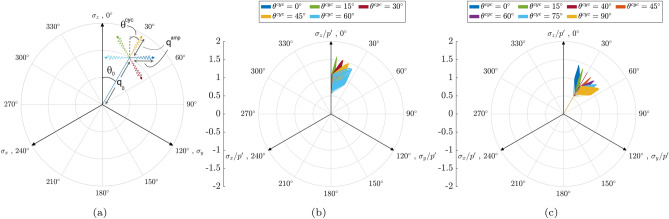
Illustration of applied stress paths for anisotropically consolidated samples under cyclic loading conditions: (**a**) schematic illustration of cyclic stress path notation in octahedral plane, (**b**) effective stress path for an anisotropically consolidated samples with $\theta _0=0^\circ$ and (**c**) effective stress path for an anisotropically consolidated samples with $\theta _0=30^\circ$.

This study simulates anisotropically consolidated samples under isobaric cyclic loading conditions, where the total mean stress remains constant throughout the loading process ($p = \text {constant}$). The loading paths consist of two stages: anisotropic consolidation, followed by undrained cyclic true triaxial loading. Initially, loading was performed under drained conditions with a constant strain rate in the *z*-direction. During this stage, the servo control mechanism was adjusted to achieve a specific Lode angle, $\theta _0$, which corresponds to the Lode angle in the anisotropic consolidation phase. This adjustment continued until the desired deviatoric stress ($q_0$) was reached (see Fig. 2a). Once the specified deviatoric stress level was achieved, the principal stress values were recorded as the initial state for the undrained cyclic loading stage. During this stage, undrained cyclic loading was applied with variations in the Lode angle, $\theta ^{cyc}$, which corresponds to the Lode angle during the cyclic shearing phase. The variations in pore water pressure and unbalanced forces on particles at the boundaries of a measurement region were also calculated. To maintain the desired cyclic Lode angle ($\theta ^{cyc}$), the stress on the upper and lower walls ($\sigma ^{\prime }_{11}=\sigma ^\prime _z$) was computed at each time step. The other two principal stresses ($\sigma ^{\prime }_{22}=\sigma ^\prime _y$ and $\sigma ^{\prime }_{33}=\sigma ^\prime _x$) were determined using isobaric conditions, excess pore water pressure values, and the servo control mechanism. Cyclic loading continued until the desired shear stress amplitude ($q^{\text {amp}}$) was reached. Subsequently, the loading direction was reversed to achieve a minimum $q^{\text {amp}} = 2$ kPa. Figure 2a illustrates the schematic of the loading conditions applied in this study, where $p_0$, $q_0$, and $\theta _0$ correspond to the consolidation phase, while $q^{\text {amp}}$ and $\theta ^{\text {cyc}}$ are related to the undrained cyclic shearing phase. This study simulates two primary loading scenarios: one involving anisotropic consolidation with $\theta _0 = 0^\circ$ followed by cyclic shearing, as depicted in Fig. 2a, and the other with anisotropic consolidation at $\theta _0 = 30^\circ$, followed by undrained cyclic shearing, as shown in Fig. 2b. The selected Lode angle values ( for preshearing phase) were chosen to replicate two real-world scenarios commonly encountered in practical applications. The anisotropically consolidated sample with $\theta _0 = 0^\circ$, representing triaxial drained conditions, illustrates the behavior of granular soils under slow, long-term loading, where excess pore pressure dissipates. In contrast, the anisotropically consolidated sample with $\theta =30^\circ$ mimics plane strain conditions, which are essential for modeling confined soil behavior in scenarios such as strip foundations, tunnels, long retaining walls, and slope stability analysis, where one dimension of deformation is negligible^51,52^. It is worth mentioning that values in Fig. 2a and b are normalized with effective mean pressure, $p^\prime$.

## Macroscale observations

### Mechanical response of anisotropically consolidated samples under undrained true triaxial loading paths

**Fig. 3:**
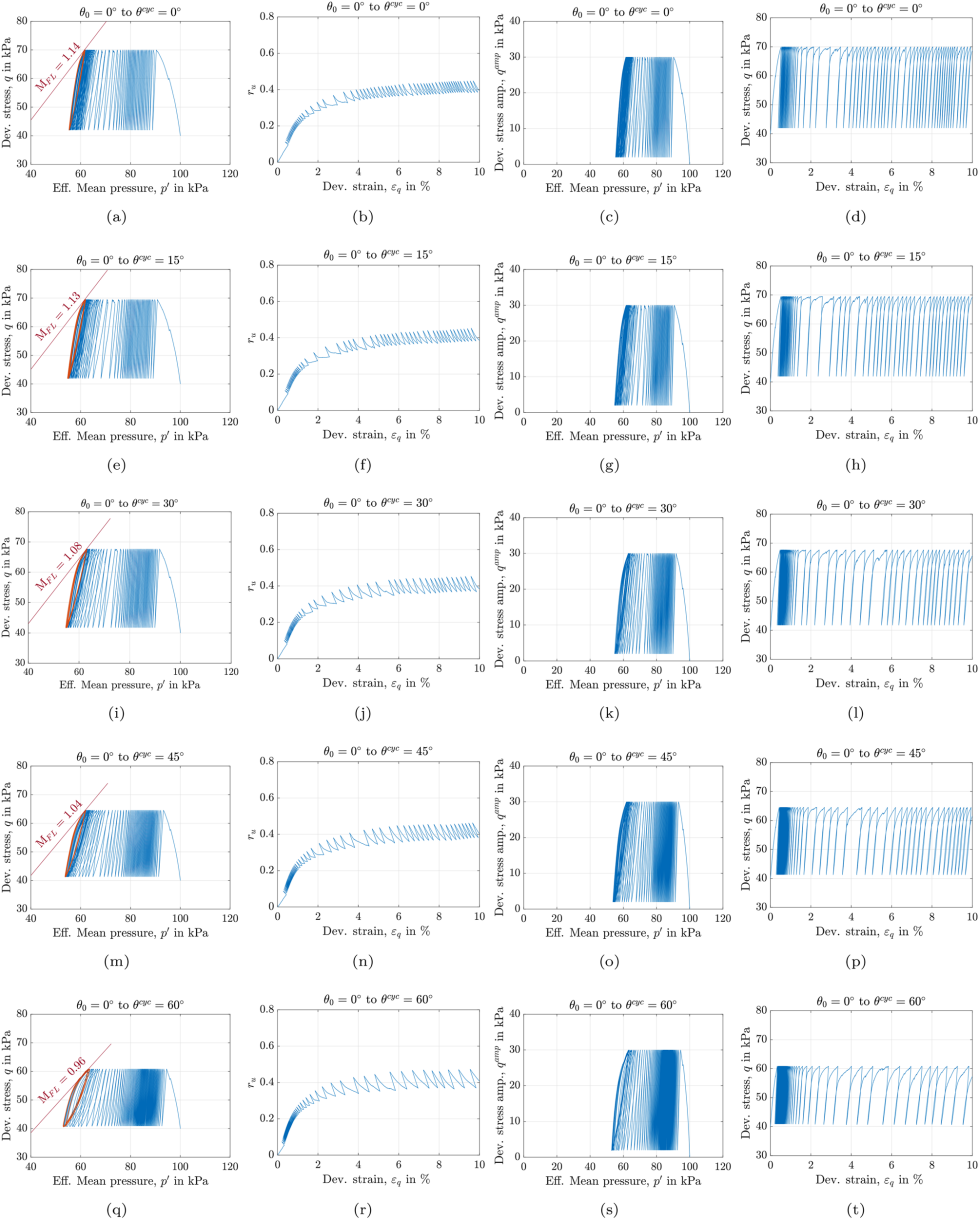
Mechanical response of anisotropically consolidated assemblies with $e_0=0.641$, $p_0^\prime =100$ kPa, $q_0=40$ kPa and $\theta _0=0^\circ$, sheared with $q^{\text {amp}}=30$ kPa and (**a**), (**b**) & (**c**): $\theta ^{cyc}=0^\circ$; (**d**), (**e**) & (**f**): $\theta ^{cyc}=15^\circ$; (**g**), (**h**) & (**i**): $\theta ^{cyc}=30^\circ$; (**j**), (**k**) & (**l**): $\theta ^{cyc}=45^\circ$ and (**m**), (**n**) & (**o**): $\theta ^{cyc}=60^\circ$.

Figure 3 illustrates the response of anisotropically consolidated assemblies with the same initial confining pressure and void ratio of $e_0=0.641$ ($D_r = 33\%$) and $p_0^\prime = 100$ kPa, sheared to reach $q_0$ = 40 kPa with a Lode angle of $\theta _0$ = 0°. From this state, undrained cyclic loading is applied at different Lode angles, $\theta ^{cyc}$, with a constant shear amplitude of $q^{\text {amp}}$ = 30 kPa. The Lode angle during the undrained cyclic loading path, $\theta ^{cyc}$, remains constant. The tests were conducted on assemblies subjected to various values of $\theta ^{cyc}$, ranging from 0° to 60°. An illustration of applied stress paths is indicated in Fig. 2b.

Independent of the Lode angle values, all samples in Fig. 3 exhibit consistent qualitative behavior: excess pore water pressure gradually increases with each loading cycle, resulting in a reduction of mean effective stress. After a certain number of cycles, the normalized excess pore water pressure, $r_u$, reaches a stable value (see, e.g., Fig. 3b). The final part of the effective stress path then forms a lens-shaped loop, which is located near the Failure Line (FL) identified from the monotonic tests (e.g., see Fig. 3a). The lens-shaped loops are also referred to as attractors. The last three loops are highlighted in orange color in Fig. 3 while the rest of the stress path is shown in blue. As shown in Fig. 3a,e,i,m,q, an increase in the Lode angle during the cyclic phase extends the number of cycles required to reach the lens-shaped loops. As the Lode angle in the cyclic phase increases from $\theta ^{cyc}=0^\circ$ to $\theta ^{cyc}=60^\circ$, the overall inclination of the lens-shaped loops decreases, and their enclosed area in the $q-p^\prime$ plane is increased. Additionally, the Lode angle influenced the mean effective stress level at which the assemblies reached the last attractor, with this stress level decreasing slightly as the Lode angle increased.

A series of undrained monotonic tests have been conducted to quantify the inclinations of the failure lines shown in Fig. 3a,e,i,m,q. Undrained monotonic loading was applied to anisotropically consolidated samples using the same Lode angle as employed in the corresponding cyclic tests. For each test, the FL is constructed by connecting the origin of the effective stress plane to the ultimate point of the effective stress path at $\varepsilon _q = 10\%$. The results show that the slope of the FL in the $q-p^\prime$ plane (M$_{\text {FL}}$) decreases as the Lode angle increases. This observation is consistent with the reduced inclination of the lens-shaped loops with increasing $\theta ^{cyc}$. As the effective stress path approaches the failure line, it starts to trace a lens-shaped loop, where both the slope of the failure line and the inclination of the loops are influenced by the Lode angle.

Figure 3b,f,j,n,r illustrate the variation of normalized pore water pressure, $r_u$, versus deviatoric strain. $r_u$ is defined as the ratio of excess pore water pressure to the initial confining pressure. It is observed that with increasing $\theta ^{cyc}$, the rate of pore water pressure accumulation decreases, i.e., pore water pressure accumulates faster in the assembly with $\theta ^{cyc} = 0^\circ$ compared to the one with $\theta ^{cyc}= 60^\circ$. It is worth noting that since $q^{\text {amp}}$ = 30 kPa holds across all tests, its effect on sample behavior is excluded from consideration in this section. As shown in Fig. 3c,g,k,o,s$, q^{\text {amp}}$ is constant during the cyclic phase, while the calculated *q* regarding the origin of the octahedral plane varies depending on the applied Lode angle.

Figure 3d,h,i,p,t show the variations of deviatoric stress, *q*, versus deviatoric strain, $\varepsilon _q$. It has been observed that the Lode angle influences the maximum deviatoric stress attained by the granular assemblies. For instance, the maximum deviatoric stress for the sample sheared with $\theta ^{cyc}=0^\circ$ is nearly 70 kPa (see Fig.3d), whereas the sample sheared with $\theta ^{cyc}=60^\circ$ reaches a maximum deviatoric stress of 60 kPa (see Fig.3t). The results indicate that, regardless of the Lode angle values, the rate of accumulated deviatoric strain decreases as the number of cycles increases. This trend is also observed in experimental data from triaxial cyclic tests on sand reported by^53^. Regarding Fig. 3d,t, the increase in deviatoric strain amplitude with the number of cycles occurs at a slower rate for the sample with $\theta ^{cyc} = 60^\circ$ compared to the one with $\theta ^{cyc} = 0^\circ$. In other words, more cycles can be applied to the sample in Fig. 3t compared to the one in Fig. 3d before reaching a specific failure criterion (here, $\varepsilon _q = 10\%$). Furthermore, the amplitude of deviatoric strain remains almost constant after assemblies reach the lens-shaped loop. It is important to note that the initial slope of the deviatoric stress-strain curve is also influenced by variations in $\theta ^{cyc}$ (see Fig. 3d and t). This effect will be further analyzed in terms of the secant shear modulus in the subsequent sections.

**Fig. 4:**
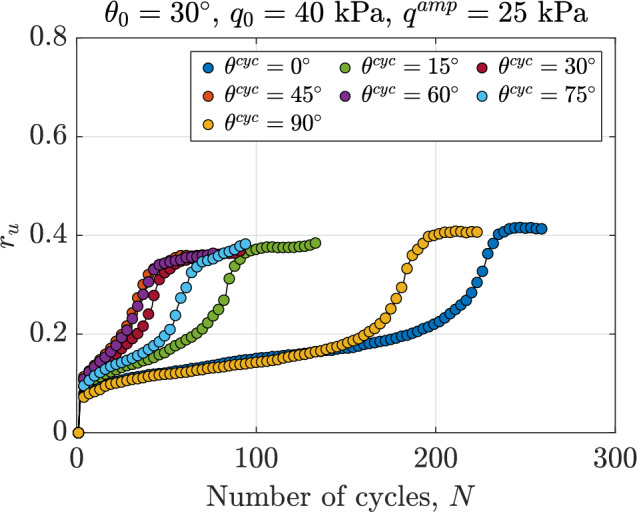
Variations in normalized excess pore water pressure versus the number of cycles for anisotropically consolidated assemblies with $e_0=0.641$, $p_0^\prime =100$ kPa, $q_0=40$ kPa and $\theta _0=30^\circ$ under undrained cyclic loading with $0^\circ \le \theta ^{\text {cyc}} \le 90^\circ$.

**Fig. 5:**
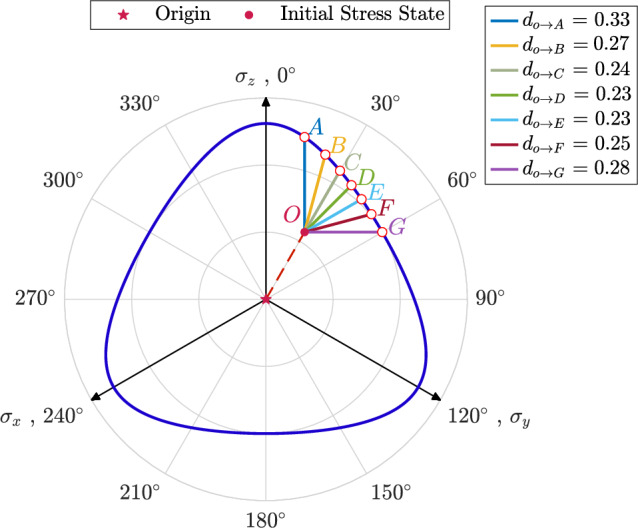
Failue surface of samples with $e_0=0.641, p_0^\prime =100\hbox { kPa}$ sheared with $q_0=25$ at $\theta _0=30^\circ$ (Of note, $d_{O\rightarrow X}$ indicates the distance from Point *O* to Point *X*).

To further analyze the effects of $\theta ^{\text {cyc}}$ on the stress path, Fig. 4 illustrates the variation in normalized excess pore water pressure, $r_u$, versus the number of cycles, *N*, for samples where $e_0=0.641$, $p_0^\prime =100$ kPa, $q_0=40$ kPa and $\theta _0=30^\circ$ subjected to shearing with $q^{\text {amp}}=25$ kPa across a broad range of $0^\circ \le \theta ^{\text {cyc}} \le 90^\circ$. An illustration of applied stress paths is demonstrated in Fig. 2c. The results reveal that samples sheared with $\theta ^{\text {cyc}}=0^\circ$ and $\theta ^{\text {cyc}}=90^\circ$ reach higher values of normalized excess pore water pressure compared to other samples. Additionally, these samples require more cycles to achieve the maximum stable value of $r_u$ compared to the others. The sequence in which the number of cycles is required to reach a stable $r_u$ for assemblies sheared with different $\theta ^{\text {cyc}}$ is as follows: $\theta ^{\text {cyc}}=0^\circ> \theta ^{\text {cyc}}=90^\circ> \theta ^{\text {cyc}}=15^\circ> \theta ^{\text {cyc}}=75^\circ> \theta ^{\text {cyc}}=30^\circ> \theta ^{\text {cyc}}=45^\circ > \theta ^{\text {cyc}}=60^\circ$. This order can be attributed to the distance between the initial stress state and the corresponding point on the failure surface, which is defined by the Lode angle. To investigate this relationship in more detail, the failure surface is defined using undrained monotonic tests based on the Lade-Duncan criteria^54^ and is illustrated in Fig. 5. The distance from the initial stress state, which is indicated as Point *O*, from the failure surface for different Lode angle values is illustrated in Fig. 5. In this sense, $d_{O\rightarrow X}$ denotes the distance from Point *O* to Point *X*. For instance, $d_{O\rightarrow A}$ is the distance from Point *O* and Point *A* and holds for samples sheared along the path with $\theta ^{\text {cyc}}=0^\circ$. Based on the results illustrated in Fig. 5: $d_{O\rightarrow A}> d_{O\rightarrow G}>d_{O\rightarrow B}>d_{O\rightarrow F}>d_{O\rightarrow C}>d_{O\rightarrow D} \ge d_{O\rightarrow E}$. It is observed that the distance from the initial state to the failure surface follows a similar order for samples with the same initial state and sheared with different values of $\theta ^{\text {cyc}}$. Regarding Fig. 5, the distance from the initial stress state to the corresponding point on the failure surface follows this order: for assembly sheared with $\theta ^{\text {cyc}}=0^\circ> \theta ^{\text {cyc}}=90^\circ> \theta ^{\text {cyc}}=15^\circ> \theta ^{\text {cyc}}=75^\circ> \theta ^{\text {cyc}}=30^\circ> \theta ^{\text {cyc}}=45^\circ > \theta ^{\text {cyc}}=60^\circ$.

### Effect of$\theta ^ {cyc}$, $\theta _0$, $q_0$ and $q^{\text {amp}}$ on accumulated pore water pressure and secant shear modulus

**Fig. 6:**
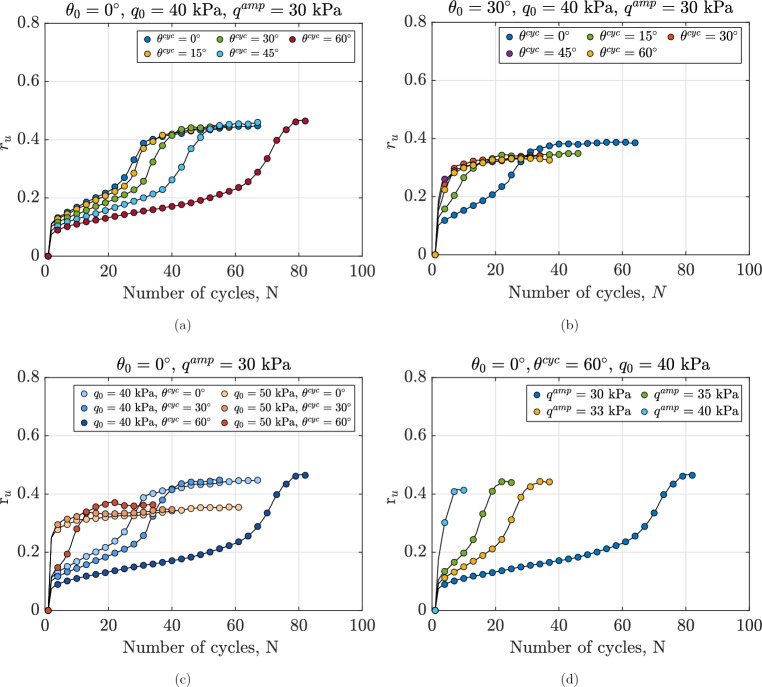
Variations in normalized excess pore water pressure for different anisotropically consolidated assemblies under undrained cyclic loading, illustrating the effects of (**a**): Lode angle in undrained cyclic phase $\theta ^{\text {cyc}}$ for samples with $\theta _0=0^\circ$, (**b**): Lode angle in undrained cyclic phase $\theta ^{\text {cyc}}$ for samples with $\theta _0=30^\circ$, (**c**) initial static shear stress $q_0$ and (**d**): deviatoric stress amplitude $q^{\text {amp}}$.

This subsection examines the variation of accumulated pore water pressure and secant modulus across various anisotropically consolidated assemblies under undrained cyclic loading. To facilitate the comparative analysis, the changes in normalized pore pressure, $r_u$, and normalized secant shear modulus versus the number of cycles, *N*, are illustrated in Figs. 6 and  9, respectively. The effects of $\theta ^{\text {cyc}}$, $\theta _0$, $q_0$, and $q^{\text {amp}}$ are discussed in detail.

#### Evolution of excess pore water pressure

Figure 6a depicts the effect of Lode angle during undrained cyclic loading $\theta ^{\text {cyc}}$ on the normalized excess pore water pressure of anisotropically consolidated samples. Tests were conducted on samples with initial conditions of $e_0=0.641$ and $p_0^\prime =100$ kPa, consolidated to $q_0=40$ kPa under a constant-p stress path with $\theta _0=0^\circ$. Undrained cyclic loading was then applied with $q^{\text {amp}}=30$ kPa at various $\theta ^{\text {cyc}}$ values. Given that all tests were conducted under identical initial stress states and subjected to the same $q^{\text {amp}}$, the analysis is exclusively focused on evaluating the influence of $\theta ^{cyc}$ during undrained cyclic loading on anisotropically consolidated samples. According to Fig. 6a, regardless of the Lode angle, $r_u$ varies with a decreasing rate until a certain number of cycles, and then the evolution rate increases to reach a stable value. It is also noted that all tests ultimately reach nearly the same maximum value of $r_u \approx 0.44$; however, as the Lode angle increases from $0^\circ$ to $60^\circ$ during cyclic loading, the number of cycles *N* required to reach the maximum value of $r_u$ also increases. As previously illustrated in Fig. 3, once the assembly attained a stable value of $r_u$, the final part of the stress path formed lens-shaped loops. It is evident that the lens-shaped loops form in the sample with $\theta ^{\text {cyc}}=0^\circ$ after approximately 40 cycles, whereas the stress path reaches the loops for the sample with $\theta ^{\text {cyc}}=60^\circ$ after about 80 cycles. With the same number of cycles, samples sheared at higher values of $\theta ^{\text {cyc}}$ exhibit lower $r_u$ values. For example, after 20 cycles, a sample with $\theta ^{\text {cyc}}=0^\circ$ achieves $r_u=0.22$, while a sample with $\theta ^{\text {cyc}}=60^\circ$ reaches $r_u=0.16$. It can be concluded that $\theta ^{\text {cyc}}$ plays a key role in the required number of cycles for samples to reach the attractor, as also illustrated for these series of tests in Fig. 3.

Figure 6b illustrates the variations of $r_u$ for samples with an initial state of $e_0=0.641$ and $p_0^\prime =100$ kPa, sheared to reach $q_0=40$ kPa under constant-p stress path with $\theta _0=30^\circ$. Then, undrained cyclic loading was applied with an amplitude of $q^{\text {amp}}=30$ kPa at different values of $\theta ^{\text {cyc}}$. An illustration of applied stress paths for this series of tests is indicated in Fig. 2c. The difference between this series of tests and the one shown in Fig. 6a is the value of $\theta _0$ in the anisotropic consolidation phase. Similar to Fig. 6a, the accumulation rate of $r_u$ is significantly influenced by $\theta ^{\text {cyc}}$. Within the first three cycles, all samples exhibit a pronounced increase in normalized excess pore water pressure. Samples with $\theta ^{\text {cyc}}=30^\circ$, $45^\circ$, and $60^\circ$ show similar rapid $r_u$ accumulation, while the sample at $\theta ^{\text {cyc}}=0^\circ$ achieves a higher $r_u$ upon reaching the failure state at $\varepsilon _q=10\%$.

**Fig. 7:**
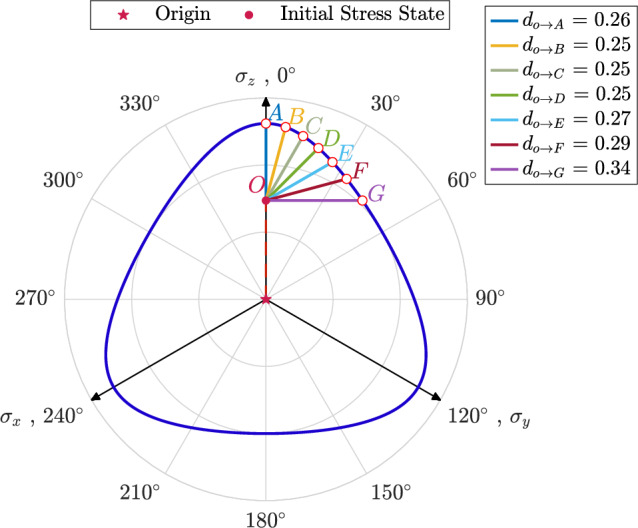
Failue surface of samples with $e_0=0.641, p_0^\prime =100\hbox { kPa}$ sheared with $q_0=25$ kPa at $\theta _0=0^\circ$ (of note, $d_{O\rightarrow X}$ indicates the distance from Point *O* to Point *X*).

A comparison between Fig. 6a and b underscores the impact of $\theta _0$ on the evolution of normalized excess pore water pressure during cyclic loading. As illustrated, the variations in $\theta _0$ heavily affect the rate of excess pore water generation in the particulate assemblies. For instance, under identical initial confining pressure and void ratio, a sample consolidated with $\theta _0 = 0^\circ$ and subjected to cyclic shear with $\theta ^{\text {cyc}} = 30^\circ$ required nearly 50 cycles to attain a maximum $r_u$ of 0.42, while a sample with $\theta ^{\text {cyc}} = 30^\circ$ and $\theta _0 = 30^\circ$ reached the maximum $r_u$ of 0.31 after only 25 cycles. It is also evident that samples subjected to the same Lode angle during both consolidation and cyclic loading phases (e.g., $\theta ^{\text {cyc}}=0^\circ$ in Fig. 6a and $\theta ^{\text {cyc}}=30^\circ$ in Fig. 6b) require a lower number of cycles to reach the lens-shaped loops. This can be attributed to the internal arrangement of particles and the reorganization of inter-particle contacts within the assembly, resulting from changes in the direction and magnitude of the applied load. It has been established in the literature that the fabric rearranges along a preferential direction that aligns with the major principal stress of the applied load^28^. This indicates that inter-particle contacts in the sample sheared with the same Lode angle as during the consolidation phase are already aligned with the major principal stress direction, requiring a higher generation of excess pore water pressure to withstand the applied load, as volumetric changes are constrained. From this perspective, the faster accumulation of $r_u$ for the sample with $\theta ^{\text {cyc}}=0^\circ$ in Fig. 6a compared to the ones with other values of $\theta ^{\text {cyc}}$ at the same strain level can be justified. The same observation applies to the assembly sheared with $\theta ^{\text {cyc}}=30^\circ$ and $\theta _0=30^\circ$ in Fig. 6b. Another factor that may influence the number of cycles required for granular assemblies to reach the maximum value of $r_u$ could be the distance between the initial stress state and the failure surface. To explore this in detail, the failure surface is defined using undrained monotonic tests based on the Lade-Duncan criteria and is illustrated in Fig. 7. For samples with $\theta _0=0^\circ$, this distance decreases in the order: $\theta ^{\text {cyc}}=0^\circ> 15^\circ> 30^\circ> 45^\circ > 60^\circ$, corresponding to the observed trend in the number of cycles required to reach the attractor state. A similar trend is observed for samples with $\theta _0=30^\circ$. From this perspective, the similarity in pore pressure accumulation for $\theta ^{\text {cyc}}$ of $45^\circ$ and $30^\circ$ in Fig. 6b can be attributed to their nearly equivalent distance from the failure surface in the direction of applied $\theta ^{\text {cyc}}$. Overall, the results indicate that the maximum value of $r_u$ is influenced by the combined effects of $\theta ^{\text {cyc}}$ and $\theta _0$.

Figure 6c illustrates the impact of initial static shear stress on behavior of samples with $e_0=0.641$ and $p_0^\prime =100$ kPa, sheared under $\theta _0 = 0^\circ$ to reach different values of $q_0$, followed by cyclic loading with $\theta ^{\text {cyc}}$ of 0°, 30° and 60°. To isolate the effect of $q_0$ for comparison, samples were sheared to reach $q_0=40$ and 50 kPa, while other factors were kept constant. Consistent with the results in Fig. 6a, regardless of the $q_0$ value, samples sheared at $\theta ^{\text {cyc}} = 0^\circ$ reach the lense-shaped loop quickly, while those sheared at 60° require more cycles to reach the attractor. The results indicate that regardless of the Lode angle, samples with a lower value of $q_0$ require more cycles to reach the attractor. Additionally, anisotropically consolidated samples with lower $q_0$ values achieved a higher maximum value of $r_u$ at the end of the tests, i.e., after reaching a deviatoric strain level of 10%. Specifically, all tests with $q_0 = 50$ kPa reached $r_u \approx 0.38$, whereas tests with $q_0 = 40$ kPa reached $r_u = 0.45$. According to the experimental data in the literature, the effect of the initial static shear stress on the accumulated pore water pressure and cyclic resistance was found to be dependent on density, mean stress, and chosen yield criteria^55–57^. The triaxial data on anisotropically consolidated samples (e.g.,^58^) indicate that for medium-dense samples with the same initial mean stress, increasing the initial deviatoric stress decreases the number of cycles required to reach liquefaction. These findings are consistent with the results presented here. The opposite trend has also been reported in experimental studies (e.g.,^5^), where an increase in static shear stress was found to enhance the liquefaction resistance of soil assemblies. These discrepancies are primarily attributed to the initial fabric anisotropy of various soil assemblies, as discussed by^6^. Investigating the effect of fabric anisotropy is not in the scope of the current study.

Figure 6d illustrates the impact of varying $q^{\text {amp}}$ on anisotropically consolidated samples with $e_0=0.641$, $p_0^\prime =100$ kPa, and $q_0 = 40$ kPa, subjected to undrained cyclic loading at $\theta ^{\text {cyc}} = 60^\circ$. For the sake of comparison, $q^{\text {amp}}$ of 30, 35, and 40 kPa were applied. It is observed that the value of $q^{\text {amp}}$ heavily affects both the number of cycles required to reach the attractor and the maximum value of $r_u$ at the end of the tests. The sample sheared with a lower amplitude of deviatoric stress requires more cycles to reach the lense-shaped loop. These findings are consistent with experimental data from triaxial tests on granular soils^53,59–61^, which show that an increase in the amplitude of the deviatoric stress results in a reduction in liquefaction resistance.

**Fig. 8:**
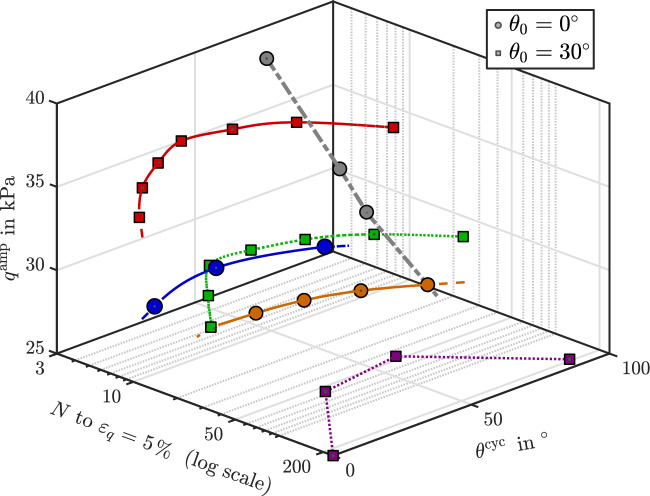
3D illustration showing the number of cycles required to reach 5% shear strain for anisotropically consolidated samples with initial angles $\theta _0$=$0^\circ$ and $\theta _0$=$30^\circ$, under varying amplitudes of deviatoric stress $q^{amp}$ and cyclic Lode angle ($\theta ^{cyc}$).

Figure 8 highlights the relationship between the number of loading cycles required to achieve 5% deviatoric strain and the applied deviatoric stress amplitude $q^{\text {amp}}$ under varying cyclic Lode angles $\theta ^{\text {cyc}}$. The results reveal a clear trend: an increase in $q^{\text {amp}}$ leads to a reduction in the number of cycles needed to reach the 5% strain level. For samples initially consolidated with $\theta _0 = 0^\circ$, the effect of $\theta ^{\text {cyc}}$ is relatively smooth and gradual. In contrast, anisotropically consolidated samples with $\theta _0 = 30^\circ$ exhibit a more pronounced sensitivity to changes in $\theta ^{\text {cyc}}$. The results highlight the interplay between the Lode direction during initial consolidation and the subsequent cyclic loading path, both of which significantly influence the number of cycles required to reach cyclic mobility.

#### Degradation of secant shear modulus

**Fig. 9:**
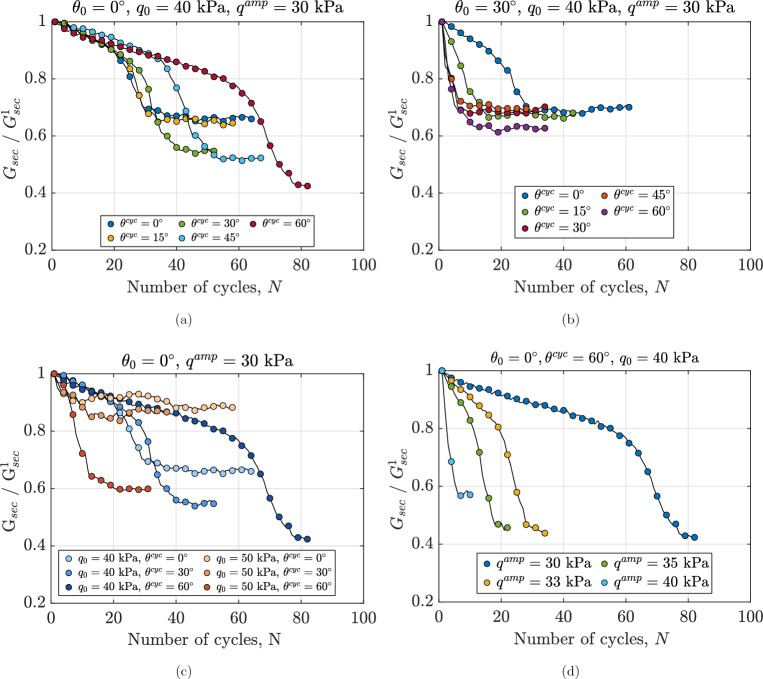
Variations in normalized secant shear modulus for different anisotropically consolidated assemblies under undrained cyclic loading, illustrating the effects of (**a**): Lode angle in undrained cyclic phase $\theta ^{\text {cyc}}$ for samples with $\theta _0=0^\circ$, (**b**): Lode angle in undrained cyclic phase $\theta ^{\text {cyc}}$ for samples with $\theta _0=30^\circ$, (**c**) initial static shear stress $q_0$ and (**d**): deviatoric stress amplitude $q^{\text {amp}}$.

This subsection describes the degradation of shear stiffness in anisotropically consolidated assemblies under true triaxial cyclic loading. To this aim, $G_\text {sec}$ is calculated at the end of each loading cycle and normalized by the secant shear modulus of the first cycle ($G^1_\text {sec}$). This normalization is crucial as it provides a dimensionless measure of stiffness degradation, allowing for direct comparison across different loading conditions and initial states. Moreover, by referencing the first cycle’s stiffness, it quantifies the relative change in material response, highlighting the progressive weakening of the granular assembly throughout the undrained cycles. Figure 9a shows the evolution of normalized secant shear modulus for assemblies with the same initial state and subjected to undrained cyclic shearing with different values of $\theta ^{\text {cyc}}$ ranging from $0^\circ$ to $60^\circ$. The macroscale response of these assemblies is also illustrated in Figs. 3 and 6a. It is evident that $\theta ^{\text {cyc}}$ influences the rate of degradation of the secant shear modulus, the minimum value of $G_\text {sec}/G^1_\text {sec}$ and the number of cycles required to reach this minimum value. The normalized secant shear modulus degrades more rapidly in the sample sheared with $\theta ^{\text {cyc}}=0^\circ$ compared to the one with $\theta ^{\text {cyc}}=60^\circ$. For the sample with $\theta ^{\text {cyc}}=0^\circ$, $G_\text {sec}/G^1_\text {sec}$ decays and reaches a near-constant value after approximately 40 cycles, with no significant changes in stiffness observed with additional undrained cycles. While for the sample with $\theta ^{\text {cyc}}=60^\circ$, degradation in stiffness continues up to around 80 cycles. A comparison of Fig. 9a and b highlights the effect of $\theta _0$ on the normalized secant shear modulus of anisotropically consolidated samples. As illustrated in Fig. 9a, the number of cycles required to achieve the minimum stiffness decreases with increasing $\theta ^{\text {cyc}}=0^\circ$ for samples sheared with $\theta _0=0^\circ$. However, Fig. 9b shows a different trend for samples with $\theta ^{\text {cyc}}=30^\circ$, which aligns with the previously described distance from the initial stress state to the failure surface. These observations highlight the critical influence of the Lode angle during the anisotropic consolidation phase.

Figure 9c illustrates the impact of initial static shear stress on the normalized secant shear modulus of anisotropically consolidated assemblies under different initial deviatoric stresses ($q_0$ = 40 kPa and 50 kPa). Lower initial static shear stress results in slower degradation of the normalized secant shear modulus for assemblies with identical initial conditions. The effect of initial static shear stress on shear modulus varies with the Lode angle ($\theta ^{\text {cyc}}$). For instance, at $\theta ^{\text {cyc}} = 60^\circ$, the sample with $q_0 = 40$ kPa exhibits a higher normalized secant shear modulus than the one with $q_0 = 50$ kPa. This trend reverses after 20 cycles for $\theta ^{\text {cyc}} = 0^\circ$ and $30^\circ$. These findings confirm that initial shear stress significantly influences the cyclic behavior and shear resistance of in-situ soil deposits. Figure 9d demonstrates that variations in deviatoric stress amplitude ($q^{\text {amp}}$) have a similar qualitative effect: higher amplitudes in undrained cyclic loading accelerate shear modulus degradation in assemblies with the same initial state.

## Micro-scale observation

In addition to analyzing macroscopic behaviors, DEM enables the study of microstructural evolution through various micro-scale quantities. In this study, the Coordination Number $\text {CN}$, redundancy index I$^f_R$, second-order inter-particle contact orientation fabric tensor, and second-order particle orientation fabric tensor are employed to describe the microstructural arrangement and packing structure of assemblies during the loading process. The microscopic observation will be used to elucidate the macro-scale response discussed in the previous sections.

### Definition of micro-scale quantities

Two different fabric tensors are employed to describe the spatial distributions of contact normals and particle orientations. In this sense, the second-order inter-particle contact normal fabric tensor $\textbf{F}^c$ and the particle orientation fabric tensor $\textbf{F}^p$ are defined as:

7 $$\begin{aligned} \textbf{F}^c = \dfrac{1}{2N_{c}}\displaystyle \sum _{k=1} ^{2N_{c}} \textbf{n}_c^k \otimes \textbf{n}_c^k \,\,\,\, ; \,\,\,\, \textbf{F}^p = \dfrac{1}{2N_{p}}\displaystyle \sum _{k=1} ^{2N_{p}} \textbf{n}_p^k \otimes \textbf{n}_p^k \end{aligned}$$

In the definition of $\textbf{F}^c$, the superscript *k* denotes the *k*th of the 2$N_{c}$ unit contact normal vectors, where *c* signifies the contacts. $\textbf{n}_c^k=\{\textbf{n}_{c1}^k \,\textbf{n}_{c2}^k\,\textbf{n}_{c3}^k\}$ denotes the direction cosine of the unit vector $\mathbf {n_c}^k$ with respect to the reference axis $x_{i}$ with $i \in \{1, 2, 3\}$. In the definition of $\textbf{F}^p$, the superscript, *k* represents the *k*th of the 2$N_{p}$ unit vectors defining long axes of $N_{p}$ particles, where $\textbf{n}_p^k=\{\textbf{n}_{p1}^k \,\textbf{n}_{p2}^k\,\textbf{n}_{p3}^k\}$ denotes the direction cosine of the unit vector $\mathbf {n_p}^k$ with respect to the reference axis $x_{i}$ with $i \in \{1, 2, 3\}$. In tandem with this, scalar measures that quantify the intensity of fabric anisotropy, denoted as $\Delta ^c$ and $\Delta ^p$, are formulated using the deviatoric parts of the fabric tensors:

8 $$\begin{aligned} \Delta ^c = \sqrt{3 \left( \textbf{F}^{c*} : \textbf{F}^{c*}\right) } \,\,\,\,;\,\,\,\, \Delta ^p = \sqrt{3 \left( \textbf{F}^{p*} : \textbf{F}^{p*}\right) } \end{aligned}$$

where $\textbf{F}^{p*}$ and $\textbf{F}^{c*}$ are the deviatoric part of $\textbf{F}^{p}$ and $\textbf{F}^{c}$, respectively. $\textbf{A}: \textbf{B}= \text{ tr }\,(\textbf{A}\cdot \textbf{B}^T) = A_{ij} B_{ij}$, wherein $\text{ tr }\,\mathbf (X) = X_{ii}$ denotes the trace of a tensor. In addition to tracking fabric development during loading, In this study, we use the redundancy index ($I^f_R$) to evaluate the stability and load-bearing capacity of the granular assembly by quantifying the excess number of contacts relative to the minimum needed for mechanical equilibrium (^62,63^. The relevant measures are defined as follows:

9 $$\begin{aligned} \textrm{CN} = \frac{\displaystyle \sum _{N_b} {n}_c^b}{{N}_p} \,\,\, ; \,\,\, I^f_{R}= \frac{(3-2f_{s})N_{c}}{6(N_{p}-N^0_{p})} \end{aligned}$$

Here, $\textrm{CN}$ denotes the coordination number, calculated as the average number of contacts per particle, where ${n}_c^b$ is the number of contacts per particle and $N_b$ is the number of bulk particles in the system. For the redundancy index $I^f_R$, $f_s$ is the sliding friction coefficient, $N_p$ is the total number of particles, and $N^0_p$ is the number of rattler particles-those with fewer than two contacts, which do not contribute significantly to the force network. The numerator $(3 - 2f_s)N_c$ represents the number of constraints imposed by frictional contacts, and the denominator $6(N_p - N^0_p)$ is the minimum number of contacts required to achieve isostatic equilibrium in a 3D system, accounting for the degrees of freedom of non-rattler particles. This definition of the redundancy index is specific to contact models that do not include rolling and twisting resistance, as used in this study. In models incorporating additional resistance mechanisms, alternative expressions for $I^f_R$ may be more appropriate (e.g.,^39,64^). For foundational background on the role of contact redundancy in granular systems, see^65^.

### Fabric evolution of anisotropically consolidated assemblies under continuous shearing

**Fig. 10:**
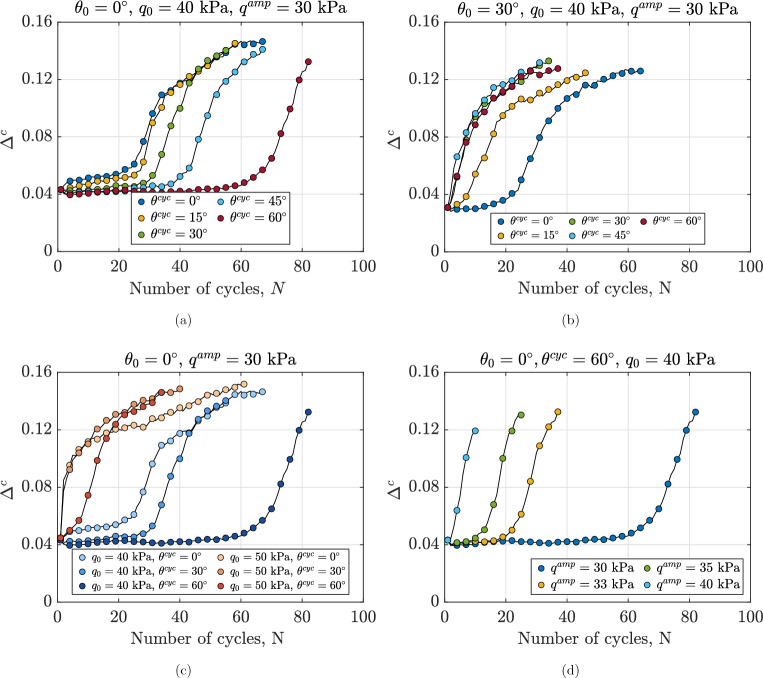
Variations in fabric invariant $\Delta ^c$ for different anisotropically consolidated assemblies under undrained cyclic loading, illustrating the effects of (**a**): Lode angle in undrained cyclic phase $\theta ^{cyc}$, (**b**): Lode angle in anisotropic consolidation phase $\theta _0$, (**c**) initial static shear stress $q_0$ and (**d**): deviatoric stress amplitude $q^{amp}$.

Figure 10 illustrates the evolution of the invariant of the inter-particle contact fabric tensor, $\Delta ^c$, for anisotropically consolidated assemblies under undrained cyclic loading. Irrespective of the Lode angle, $\Delta ^c$ exhibits a gradual increase followed by an abrupt rise to a maximum value. The initial gradual increase corresponds to the progressive build-up of excess pore water pressure during the first loading cycles, while the sudden spike coincides with the rapid increase in $r_u$ preceding the formation of lens-shaped loops (as shown in Fig. 3). This evolution of $\Delta ^c$ reflects the dynamic nature of inter-particle contacts, involving the formation of new contacts, loss of existing ones, and reorientation of contact directions. The observed trend aligns with previous research by^14^, which noted that while most contacts persist throughout loading, their orientations continuously change. During the formation of lens-shaped loops, the minimum mean effective stress allows for easier disruption, creation, and reorientation of contacts, contributing to the continuous increase in fabric invariant. This behavior is consistent with the established understanding that contacts gradually align with the major principal stress direction during shearing. In this context, contacts oriented along the major principal stress direction tend to bear higher forces, while those aligned with the minor principal stress direction carry lower forces^66–68^. The influence of the Lode angle during the cyclic phase ($\theta ^{\text {cyc}}$) on the evolution of $\Delta ^c$ is evident in Fig.  10a. Under identical loading conditions and cycle numbers, samples with $\theta ^{\text {cyc}}=0^\circ$ exhibit higher $\Delta ^c$ values compared to those with $\theta ^{\text {cyc}}=60^\circ$. This suggests that contacts in the latter sample require more contact rearrangement to align with the principal stress direction, resulting in more dilative behavior and the formation of a structure better able to withstand applied loads. This interpretation is supported by the higher normalized secant shear modulus observed for $\theta ^{\text {cyc}}=60^\circ$ in Fig. 9a. Figure 10b–d demonstrate the dependence of $\Delta ^c$ evolution on various factors. Comparison of Fig. 10a and b reveals that the rate of inter-particle contact fabric evolution is influenced by the Lode angle during the anisotropic consolidation phase. Figure 10c and d indicate clear dependencies of the evolution rate and pattern of the inter-particle fabric invariant on initial static shear stress ($q_0$) and deviatoric stress amplitude ($q^{\text {amp}}$). Notably, Fig. 10c shows that an increase in $q_0$ from 40 kPa to 50 kPa accelerates the rate of change in $\Delta ^c$ for assemblies with the same initial state. This acceleration corresponds to a lower normalized shear secant modulus and a higher rate of pore water pressure accumulation, further elucidating the complex interplay between micro-scale fabric evolution and macro-scale mechanical behavior in granular materials under cyclic loading.

**Fig. 11:**
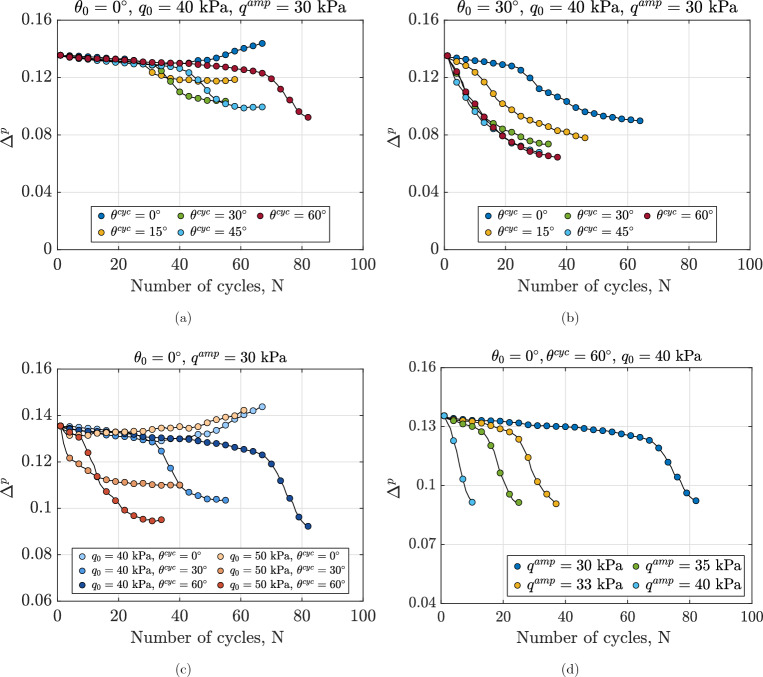
Variations in fabric invariant $\Delta ^p$ for different anisotropically consolidated assemblies under undrained cyclic loading, illustrating the effects of (**a**): Lode angle in undrained cyclic phase $\theta ^{\text {cyc}}$, (**b**): Lode angle in anisotropic consolidation phase $\theta _0$, (**c**) initial static shear stress $q_0$ and (**d**): deviatoric stress amplitude $q^{\text {amp}}$.

Figure 11 demonstrates the variations in particle orientation fabric invariant $\Delta ^p$ across different anisotropically consolidated assemblies under undrained cyclic loading. Figure 11a shows the effect of $\theta ^{\text {cyc}}$ for assemblies with the same initial stress state and void ratio. The fabric anisotropy of particle orientation shows minimal variation before the 25th cycle due to the limited deformation of the specimen. Beyond this point, the degree of anisotropy either decreases or increases with the number of cycles, depending on the value of $\theta ^{\text {cyc}}$. As deformation increases during continuous shearing, more particles rotate their long axes toward the loading direction, leading to changes in both the particle orientation fabric tensor and its invariant. The decrease in $\Delta ^p$ suggests that the particle orientation fabric of the samples becomes more isotropic by the end of the tests. Figure 11b–d show the role of $\theta _0$, $q_0$ and $q^{\text {amp}}$ on the evolution pattern and minimum value of $\Delta ^p$ across various assemblies. The results highlight that $\theta _0$ and $q_0$ primarily influence the rate of change in $\Delta ^p$, as they also affect the rate of decay in normalized secant modulus and normalized excess pore water pressure in Figs. 6b,c and  9b,c. Moreover, $q^{\text {amp}}$ affects the evolution rate, evolution pattern, and final value of the particle orientation fabric. In general, the results confirmed that the anisotropy induced by the applied load is significantly affected by the Lode angle during both the anisotropic consolidation phase and undrained cyclic loading, as well as by the initial static shear stress and the amplitude of the loading cycles.

### Variations of coordination number and redundancy index during continuous shearing

**Fig. 12:**
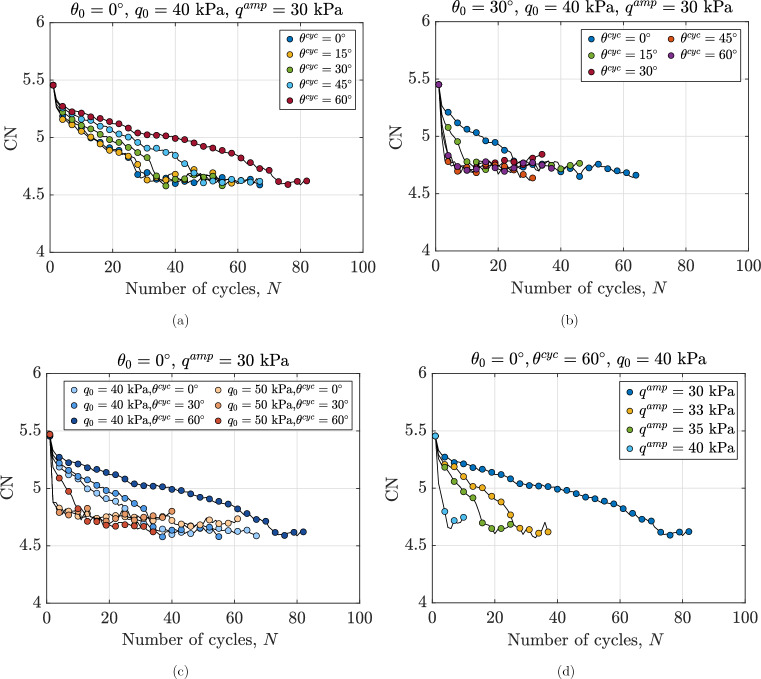
Variations in coordination number for different anisotropically consolidated assemblies under undrained cyclic loading, illustrating the effects of (**a**): Lode angle in undrained cyclic phase $\theta ^{\text {cyc}}$, (**b**): Lode angle in anisotropic consolidation phase $\theta _0$, (**c**) initial static shear stress $q_0$ and (**d**) deviatoric stress amplitude $q^{\text {amp}}$.

Figure 12a shows the evolution of coordination number for assemblies with the macro-scale response shown in Fig. 3. The coordination number represents the average number of contacts each particle has with its neighbors, providing insight into the degree of interlocking within the assembly. Regarding Fig. 12a, the CN of particles decreases from the first loading cycles and finally converges to an ultimate value with an increase in the number of cycles. This ultimate stable value is reached when the lens-shaped loops have been formed as illustrated in Fig. 3. Regardless of the Lode angle or initial conditions, all samples reach a minimum CN of 4.6 at the end of the tests when $\varepsilon _q=10 \%$ is reached. Previous studies mainly consider particles with two or more contacts in the definition of mechanical coordination number (MCN) and documented a value of MCN$=3$ (e.g., 71) or MCN$=3.2$ (e.g.,^64^) at the liquefied state of a granular assembly. For granular assemblies composed of spherical particles, a CN of 4 has been proposed as the threshold marking the transition from solid-like to fluid-like behavior^43,69^. In the simulations presented in Fig. 12, all samples exhibit a CN greater than 4, indicating that the system can be considered as solid-like. This is consistent with the macro-scale observations, as the samples do not reach a state of liquefaction in these tests. As shown in Fig.12a, CN decreases with smaller oscillations for assemblies sheared at a higher Lode angle which can be related to the evolution rate of pore water pressure. In these cases, the normalized excess pore pressure accumulates more slowly compared to the others, as shown in Fig. 6a. It is also observed that samples sheared with a higher magnitude of Lode angle have higher CN; which typically correlates with the greater shear stiffness of the assemblies shown in Fig. 9a. A comparison between Fig. 12a and b shows that the reduction rate of CN and the number of cycles required to reach the minimum CN are highly dependent on $\theta _0$ and $\theta ^{\text {cyc}}$.

**Fig. 13:**
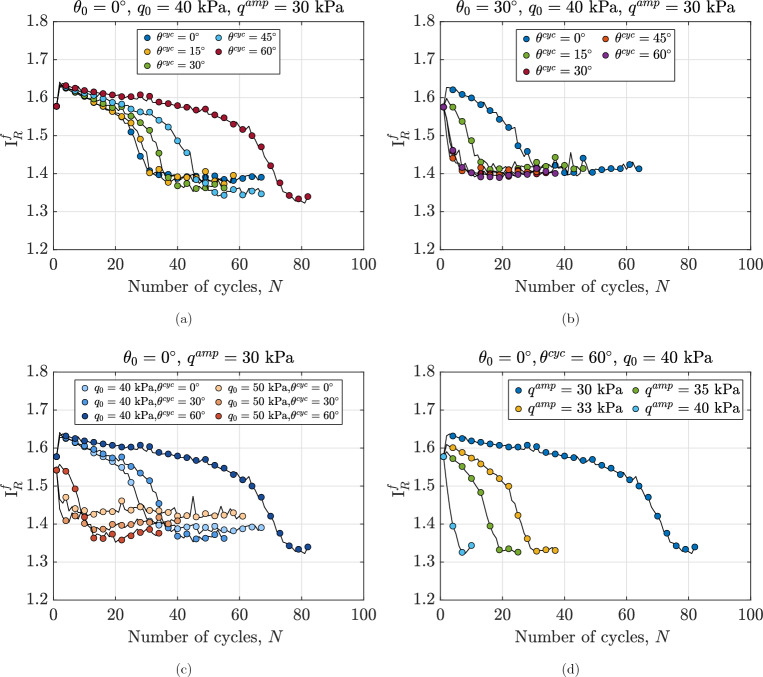
Variations in redundancy index for different anisotropically consolidated assemblies under undrained cyclic loading, illustrating the effects of (**a**): Lode angle in undrained cyclic phase $\theta ^{\text {cyc}}$, (**b**): Lode angle in anisotropic consolidation phase $\theta _0$, (**c**) initial static shear stress$q_0$ and (**d**): deviatoric stress amplitude $q^{\text {amp}}$.

In the absence of gravity, several particles float and do not contribute to the overall force transmission during the shearing process. These particles are typically known as rattlers or floaters. The variations in the redundancy index are considered to exclude the floater particles from consideration. The evolution of the redundancy index of the assemblies with macro-scale responses shown in Fig. 3 is presented in Fig. 13a. The redundancy index of the samples increases from its initial value during the first cycle, consistent with the maximum shear modulus observed in this cycle. After applying subsequent cycles, the redundancy index decreases until it reaches an ultimate value. Regardless of the Lode angle, all assemblies reach nearly the same minimum redundancy index, which is greater than 1. However, assemblies with a higher Lode angle require more cycles to achieve this minimum. The same behavior is observed in Fig. 13b. Overall, I$^f_R>1$ highlights that the assembly is consistently hyperstatic and stable^71^ and I$^f_R=1$ is mainly reported for the onset of liquefaction^70^. Moreover, the highest number of cycles required to reach the minimum redundancy index for samples sheared with $\theta ^{\text {cyc}}=0^\circ$ is compatible with the higher number of cycles needed to reach the maximum excess pore water pressure in Fig. 6a and the minimum $G_\text {sec}/G^1_\text {sec}$ in Fig. 9a. During each mechanical cycle, some contacts are lost while new ones form. The overall stability of the sample is undermined when a substantial portion of these contacts is lost, highlighted by an abrupt reduction in I$^f_R$.

Figures 12c,d and 13c,d illustrate the dependence of I$^f_R$ and CN on the initial static shear stress and the deviatoric stress amplitude. By comparing the macroscale responses in Figs. 6c,d and 9c,d with the microscale entities in Figs. 12c,d and 13c,d, it can be concluded that a higher rate of stiffness degradation corresponds to a higher rate of excess pore water pressure generation. This is due to an abrupt reduction in the average number of contacts per particle (indicated by a decrease in CN) and a reduction in the number of contacts participating in force transmission (indicated by a decrease in I$^f_R$). Moreover, the rates of decay in CN and I$^f_R$ increase with increasing $q_0$ and $q^{\text {amp}}$.

## Summary and conclusions

This study has provided micro- to macro-scale insights into the behavior of anisotropically consolidated granular assemblies subjected to undrained true triaxial loading paths. By conjugating the DEM with the CFM, the fluid-solid interaction under undrained conditions was captured. The examined loading paths included two stages: first, anisotropic consolidation along a constant-p path, and second, undrained cyclic loading. During consolidation, samples were sheared at different Lode angles to establish specific initial static shear stresses. Subsequently, undrained cyclic loading was applied with constant shear stress amplitudes across various Lode angles. Additionally, the microstructural evolution was examined within the assemblies through the coordination number, redundancy index, inter-particle contact fabric, and particle orientation fabric tensor. The following results have been obtained:

- The results indicated that the Lode angle values during cyclic loading impact the secant shear modulus, the number of cycles required to reach the lens-shaped loops, and the generation of pore water pressure. This trend was mainly attributed to the alignment of inter-particle contacts with the direction of the major principal applied stress as well as the distance between the initial state and the failure surface.
- The micro-scale observations revealed that the fabric of assemblies aligns along the preferential direction corresponding to the major principal stress, influencing the dilative response. Moreover, a lower rate of excess pore water pressure accumulation and a higher secant shear modulus are associated with samples exhibiting a slower rate of increase in invariant of inter-particle contact fabric tensor by increasing the number of cycles.
- The results also confirm that the higher rate of stiffness degradation during continuous shearing is associated with a sharp decline in both the average number of contacts per particle and the number of contacts contributing to force transmission.
- The simulations indicated that the initial shear stress across in-situ soil deposits can significantly affect their subsequent cyclic behavior and shear resistance. Regardless of Lode angle values, higher amplitudes of deviatoric stress in undrained cyclic loading accelerate the loss of shear modulus in assemblies with the same initial state.

## Data Availability

The datasets and materials used in this study are available from the corresponding author upon request.
